# Quantification of missing prescriptions in commercial claims databases: results of a cohort study

**DOI:** 10.1002/pds.4165

**Published:** 2017-01-25

**Authors:** Maria Soledad Cepeda, Daniel Fife, Michel Denarié, Dan Bradford, Stephanie Roy, Yingli Yuan

**Affiliations:** ^1^ Department of EpidemiologyJanssen Research and Development, LLCTitusvilleNJUSA; ^2^ IMS Real World Evidence SolutionsPlymouth MeetingPAUSA

**Keywords:** claims databases, limitations of observational studies, misclassification of exposure, pharmacoepidemiology

## Abstract

**Purpose:**

This study aims to quantify the magnitude of missed dispensings in commercial claims databases.

**Methods:**

A retrospective cohort study has been used linking PharMetrics, a commercial claims database, to a prescription database (LRx) that captures pharmacy dispensings independently of payment method, including cash transactions. We included adults with dispensings for opioids, diuretics, antiplatelet medications, or anticoagulants. To determine the degree of capture of dispensings, we calculated the number of subjects with the following: (1) same number of dispensings in both databases; (2) at least one dispensing, but not all dispensings, missed in PharMetrics; and (3) all dispensings missing in PharMetrics. Similar analyses were conducted using dispensings as the unit of analysis. To assess whether a dispensing in LRx was in PharMetrics, the dispensing in PharMetrics had to be for the same medication class and within ±7 days in LRx.

**Results:**

A total of 1 426 498 subjects were included. Overall, 68% of subjects had the same number of dispensings in both databases. In 13% of subjects, PharMetrics identified ≥1 dispensing but also missed ≥1 dispensing. In 19% of the subjects, PharMetrics missed all the dispensings. Taking dispensings as the unit of analysis, 25% of the dispensings present in LRx were not captured in PharMetrics. These patterns were similar across all four classes of medications. Of the dispensings missing in PharMetrics, 48% involved a subject who had >1 health insurance plan.

**Conclusions:**

Commercial claims databases provide an incomplete picture of all prescriptions dispensed to patients. The lack of capture goes beyond cash transactions and potentially introduces substantial misclassification bias. © 2017 The Authors. *Pharmacoepidemiology & Drug Safety* Published by John Wiley & Sons Ltd.

## Introduction

Accurate classification of drug exposure is crucial in observational studies. Random misclassification of the exposure leads to error in estimating the association between drug exposure and outcome measures. However, claims databases do not capture dispensings where the patient fails to use a pharmacy benefit, such as when paying cash. Thus, the extent of the lack of capture of dispensings may have increased when Wal‐Mart started a program in 2006 in which generic prescription drugs were sold for just $4 per 30‐day supply and $10 for a 90‐day supply.^1^ Claims databases may not capture dispensings through such programs. Individuals with health insurance may opt to pay out of pocket for these dispensings to save money, because the copayment may be more than $4.^2^, ^3^ Because insurance adjudication of these transactions is not needed, pharmacy claims may not be submitted for reimbursement, and these dispensings are likely to be absent in health insurance plan claims databases. Programs like this have expanded dramatically, and currently, many pharmacies offer similar programs. Furthermore, there may be many other reasons for lack of capture of dispensings such as the use of vouchers and assistance programs.

Claims databases are currently used for a wide variety of research purposes and are an important source of real world evidence. They have frequently been used to characterize utilization patterns, track patient outcomes, and conduct formal pharmacoepidemiologic evaluation studies.^4^ Incomplete capture of medication dispensings in claims databases may lead to misclassification of the exposure, errors in calculating patient adherence, and incorrect estimation of the safety of medications.

We therefore sought to estimate the magnitude of missed dispensings in a commercial claims database across four commonly prescribed therapeutic categories.

## Methods

To assess the magnitude of the lack of capture, we identified a commercial claims database (IMS Health Real‐World Data Adjudicated Claims), from now on termed PharMetrics Plus, that could be linked to a pharmacy database that captures all transactions, including cash transactions (IMS Health Real‐World Data Longitudinal Prescriptions), from now on termed LRx. We conducted a retrospective cohort study.

### IMS Health Real‐World Data Adjudicated Claims (PharMetrics Plus)

The PharMetrics Plus database holds pharmacy, provider, and facility claims for approximately 150 million patients enrolled in US health insurance plans, with an annual capture of ~40 million. Of these patients, 97% are commercially insured, 2% have Medicare Advantage coverage, and 1% have Medicaid coverage. The health insurance plans included have a wide geographic US representation. PharMetrics Plus is representative of the US commercially insured population for individuals under age 65 years.

### IMS Health Real‐World Data Longitudinal Prescriptions (LRx)

LRx is a longitudinal prescription database that covers 88% of all retail dispensing in the USA, with robust coverage in all states. From each of the pharmacies in its panel, LRx captures all dispensings. Dispensings are included whether paid for by insurance or entirely by the consumer. All forms of payment are fully represented: cash, Medicaid, Medicare Part D, and commercial insurance plans. LRx includes prescription data from a variety of outpatient pharmacies, including chains, food stores, mass merchandisers, and independent stores across the USA.

In addition to information on the medication dispensed, quantity dispensed, and days' supply of the dispensing, LRx also includes age, gender and 3‐digit zip code of the patient, specialty of the prescriber, payment type, and the use of co‐pay card or other vouchers.

Both LRx and PharMetrics Plus capture over‐the‐counter medications dispensed by the pharmacy through a prescription, and neither captures over‐the‐counter medications dispensed without a prescription.

These two IMS databases, PharMetrics Plus and LRx, are linked by IMS at the individual patient level using a multi‐level matching algorithm based on 14 encrypted data elements that include gender, date of birth, last name, first name, address, city, state, and zip code. The algorithm for identifying matched patients considers the completeness of the attributes as well as the number of variables on which patients match. Data fields with missing elements can be omitted at each match level so that patients with incomplete information can still be matched based on those patient attributes that are present. IMS Health's system is robust to problems introduced by missing data, typographical data entry error, last name changes, and change of patients' addresses. IMS Health estimates a false positive rate of 1–2% and a false negative rate of approximately 3.5%.

Both databases went through the IMS standard quality control and adjudication processes to ensure that the data transactions were considered final and that they could be used for research purposes.

## Inclusion and Exclusion Criteria

Subjects in PharMetrics Plus with continuous enrollment with medical and pharmacy benefits from 1 April 2014 to 31 March 2015 who were linked to the LRx database were included.

We excluded subjects whose pharmacies were not contributing data constantly to the LRx database for the time frame, those whose start date in the LRx database was after April 2014, and those with multiple matching patient IDs.

Once the link was established, all the pharmacy claims for four medication classes present in the LRx database from 1 April 2014 to 31 March 2015 were obtained. The four medication classes were opioid, diuretic, antiplatelet medications, and anticoagulants. These medication classes were selected because they are commonly prescribed and have a high dispensing rate of generic formulations. The list of codes used to identify the four medication classes is included in Table S1.

## Analysis

Both databases included some duplicate dispensings, and these were removed at the patient‐NDC‐Date‐Quantity level.

To determine the degree of capture of dispensings in PharMetrics Plus, we calculated for each of the four medication classes:

1. The number of subjects with the same number of dispensings in both databases, meaning that PharMetrics Plus captured all the dispensings present in LRx
2. The number of subjects with at least one dispensing in PharMetrics Plus but fewer dispensings in PharMetrics Plus than in LRx, meaning that at least one dispensing was captured and at least one dispensing was missed in PharMetrics Plus, and
3. The number of subjects for whom all dispensings were missed in PharMetrics Plus.

To assess whether a dispensing in LRx was present in PharMetrics Plus, we needed to have the dispensing in PharMetrics Plus to be for the same medication class and within ±7 days in LRx to allow for administrative system delays.

For the overall analysis by subject, subjects who had dispensings for more than one medication class were counted for each medication class they received; therefore, they were counted more than once when we provided overall results.

In addition, we conducted a similar analysis in which the unit of analysis was dispensings, not subjects. We report overall results by medication class.

LRx and PharMetrics Plus are not perfect systems, and both miss dispensings, so neither is a gold standard; nonetheless, the comparison of the number of dispensings captured in one and missing from the other is informative and analogous to the capture–recapture models that develop estimates by comparing two incomplete sources of data.^5^, ^6^ Patients with missing dispensings in the LRx database, that is, there were more dispensings in PharMetrics Plus, were not analyzed, as the aim of the study was to quantify the amount of missed capture of dispensings in claims databases. It is expected that some dispensings are not present in LRx database because it does not cover 100% of retail pharmacies.

To understand the reasons for lack of capture of dispensings in PharMetrics Plus, we stratified the results by age, gender, the number of health insurance plans the subject had, and type of payment of the dispensings. Type of payment included cash, commercial insurance, Medicaid, Medicare, and the use of vouchers or discount programs. The number of health insurance plans for each subject was obtained from LRx. In LRx, each dispensing has a plan ID and a payer ID. We concatenated the two IDs and counted the number of health insurance plans per patient.

In addition, we built a logistic regression model in which the outcome was subjects with all their dispensings missing in PharMetrics Plus compared with subjects with none of their dispensings missing in PharMetrics Plus. To assess the association of age, gender, having more than one plan, and frequency of cash payment with having all the prescriptions missing in PharMetrics Plus while controlling for the medication class, we included these other variables as covariates in the logistic regression model.

Age and cash payment were categorical variables. Age was grouped into three categories—up to 44, 45 to 64, and 65 years or older. Cash payments were grouped into three categories as well—none of the dispensings were paid in cash, up to 49% of the dispensings were paid in cash, and 50% or more of the dispensings were paid in cash.

To assess the impact of misclassification of the exposure on the estimate of the association of the exposure with the outcome, we conducted a hypothetical deterministic sensitivity analysis (simple bias analysis) assuming that the misclassification of the exposure (drug versus no drug) was independent of the outcome.^7^ We assumed that the exposure doubled the risk of the outcome.

STATA SE version 12.1 was used to conduct the analyses.

## Results

The flow of subjects is described in Table S2. A total of 1 426 498 subjects met the inclusion criteria, 56% were women, 44% were 55 or older, 57% had dispensings of opioids, 30% of diuretics, 7% of antiplatelet medications, and 6% of anticoagulants. Fifteen percent of subjects had more than one health insurance plan (Tables 1 and 2).

**Table 1:** Characteristics of subjects and number of subjects with incomplete data in PharMetrics Plus

	Total number of subjects (Col. %)	Number of subjects with the same number of dispensings in LRx and PharMetrics Plus (Col. %)	Number of subjects with some matched dispensing and ≥1 dispensings missing in PharMetrics Plus (Col. %)	Number of subjects with no dispensings in PharMetrics Plus (Col. %)
Number of subjects	1 426 498	972 116	187 749	266 633
Age in years				
<18	33 261 (2)	27 407 (3)	985 (1)	4869 (2)
18–34	179 907 (13)	135 098 (14)	15 741 (8)	29 068 (11)
35–44	208 827 (15)	150 055 (15)	26 890 (14)	31 882 (12)
45–54	367 097 (26)	253 558 (26)	53 933 (29)	59 606 (22)
55–64	491 368 (34)	335 476 (34)	73 371 (39)	82 521 (31)
65+	146 038 (10)	70 522 (7)	16 829 (9)	58 687 (22)
Gender				
Women	801 560 (56)	551 502 (57)	103 790 (55)	146 268 (55)
Men	624 938 (44)	420 614 (43)	83 959 (45)	120 365 (45)
Plans				
1	1 204 967 (84)	926 425 (95)	70 068 (37)	208 474 (78)
>1	221 531 (15)	45 691 (5)	117 681 (63)	58 159 (22)

**Table 2:** Number of subjects in each medication class and proportion of subjects with missing dispensings in PharMetrics Plus

Drug class	Number of subjects in LRx and PharMetrics Plus	Number of subjects with same number of dispensings in LRx and PharMetrics Plus	Number of subjects with some matched dispensing and ≥1 dispensings missing in PharMetrics Plus	Number of subjects with no dispensings in PharMetrics Plus
Diuretics (row %)	426 696	257 451 (60)	89 339 (21)	79 906 (19)
Opioids (row %)	821 205	620 646 (76)	63 732 (8)	136 827 (17)
Antiplatelet (row %)	96 626	48 080 (50)	17 481 (18)	31 065 (32)
Anticoagulants (row %)	81 971	45 939 (56)	17 197 (21)	18 835 (23)

Overall, 972 116/1 426 498 (68%) of subjects had the same number of dispensings in both databases. In 187 749/1 426 498 (13%) of the subjects, PharMetrics Plus captured at least one dispensing, but missed at least one dispensing, and in 266 633/1 426 498 (19%) of subjects, PharMetrics Plus missed all the dispensings (Table 1).

The gender of the subject was not associated with the capture of dispensings in PharMetrics Plus (Table 1). In contrast, age was associated with the degree of capture of dispensings in PharMetrics Plus. PharMetrics Plus missed all dispensings for 19% of subjects overall, and missed all the dispensings in 58 687/146 038 (40%) of subjects 65 years and older.

The logistic regression results showed the following: (1) Subjects aged 65 years and older had higher odds of having all their dispensings missing in PharMetrics Plus than subjects 44 or younger, OR = 3.83 (95%CI 3.78 to 3.89); (2) subjects with >1 health insurance plan also had higher odds of having all of their dispensings missed in PharMetrics Plus than subjects with only one health insurance plan, OR = 1.47 (95%CI 1.45 to 1.49); and (3) subjects who paid 50% or more of their dispensing in cash had higher odds of having all their dispensings missing in PharMetrics Plus than subjects who never paid in cash, OR = 13.97 (95%CI 13.70 to 14.25).

In terms of the different medication classes, the capture of dispensing for opioids in PharMetrics Plus was higher than for the other medications, but the pattern was similar for all the medications (Table 2).

In the analysis where the unit of analysis was the dispensings, 1 621 054/6 594 154 (25%) of the dispensings present in LRx database were not captured in PharMetrics Plus (Table 3).

**Table 3:** Number of dispensings in each medication class and number of missing dispensings in PharMetrics Plus

	Number of dispensings in LRx	Same number of dispensings in LRx and PharMetrics Plus	More dispensings in LRx than PharMetrics Plus
Diuretics (row %)	2 567 019	1 966 408 (77)	600 611 (23)
Opioids (row %)	2 944 767	2 223 703 (76)	721 064 (24)
Antiplatelet (row %)	572 901	409 375 (72)	163 526 (28)
Anticoagulants (row %)	509 467	373 614 (73)	135 853 (27)
Total	6 594 154	4 973 100 (75)	1 621 054 (25)

A large number of dispensings not captured in PharMetrics Plus were self‐paid or paid by Medicare (Table 4).

**Table 4:** Number of dispensings not captured in PharMetrics Plus by type of payment and medication class

	Diuretic Dispensings in LRx (Col %)	Diuretic dispensings not captured in PharMetrics (Col %)	Opioid Dispensings LRx (Col %)	Opioid dispensings not captured in PharMetrics (Col %)	Antiplatelet dispensings LRx (Col %)	Antiplatelet dispensings not captured in PharMetrics (Col %)	Anticoagulant dispensings LRx (Col %)	Anticoagulant dispensings not captured in PharMetrics (Col %)	Total dispensings not captured in PharMetrics (Col %)
Number of dispensings	2 567 019	600 611	2 944 767	721 064	572 901	163 526	509 467	135 853	1 621 054
Self‐paid	122 311 (5)	106 424 (18)	85 839 (3)	66 163 (9)	43 309 (7)	36 638 (22)	21 630 (4)	17 261 (13)	226 486 (14)
Medicaid	7716 (0)	7187 (1)	21 537 (0.6)	19 354 (3)	3799 (1)	3661 (2)	1842 (0)	1669 (1)	31 871 (2)
Medicare	139 283 (5)	124 453 (21)	193 340 (7)	174 253 (24)	44 664 (8)	41 532 (25)	45 809 (9)	42 762 (31)	383 000 (24)
Commercial insurer	2 297 709 (89)	362 547 (60)	2 644 051 (90)	461 294 (64)	481 129 (84)	81 695 (50)	440 186(86)	74 161 (55)	979 697 (60)

Of the dispensings missed in PharMetrics Plus and paid by a commercial insurer, 474 307/979 697 (48%) involved a subject who had >1 health insurance plan (Table 5). Although there is a relatively small number of dispensings that are covered by vouchers, discount cards, federal or state assistance programs, or workers compensation, 115 334/152 520 (76%) of these dispensings are not captured in PharMetrics Plus, and they accounted for 12% (115 334/979 697) of the dispensings missed in PharMetrics Plus (Table 5).

**Table 5:** Number of dispensings covered by commercial insurers present and missing in PharMetrics Plus by reasons

Dispensings covered by commercial insurers	Number of dispensings covered by commercial insurers in LRx (%)	Number of dispensings present in PharMetrics Plus (%)	Number of dispensings missing in PharMetrics Plus (%)
Total number of dispensings	5 863 075 (100)	4 883 378 (100)	979 697 (100)
Number of health insurance plans			
1	4 362 558 (74)	3 857 168 (79)	505 390 (52)
>1	1 500 517 (26)	1 026 210 (21)	474 307 (48)
Vouchers/discount cards/assistant programs/workers compensation			
Yes	152 520 (3)	37 186 (1)	115 334 (12)
No	5 710 555 (97)	4 846 192 (99)	684 363 (88)

## Sensitivity Analysis

A cohort study conducted in a claims database might be expected to misclassify 20% of exposed subjects as unexposed. We sought to estimate the impact of that on the risk ratio estimate in a cohort study in which the true risk ratio was 2, and there were equal numbers of exposed and non‐exposed subjects, and the misclassification was non‐differential. With this misclassification, the risk estimate was reduced to 1.7 (Table 6), which is equivalent to a 15% bias ((2 – 1.7/2) * 100). A 40% misclassification, as was identified among subjects aged 65 years and older, produced 25% bias, that is, a relative risk estimate of 1.5.

**Table 6:** Sensitivity analysis to assess the impact of a 20% of exposed subjects misclassified as non‐exposed

Truth	Exposed	Not exposed	Total	Relative risk 95%CI	Observe after misclassification	Exposed	Not exposed	Total	Biased relative risk 95%CI
Event	56	28	84	2	Event	45	39	84	1.73
No event	344	372	716		No event	275	441	716	
Total	400	400	800		Total	320	480	800	

The misclassification is assumed to be independent of the outcome

## Discussion

Commercial claims databases fail to capture a substantial number of dispensings, and in 19% of the subjects, they fail to capture all dispensings. This substantial loss of drug exposure information is a source of misclassification bias in studies using commercial claims databases. The sensitivity analysis showed that, for studies in which the number of unexposed and exposed subjects is similar, the underestimation of the association between the exposure and the outcome can be considerable, around 15%, and even larger in studies assessing subjects aged 65 years and older, a group in which the lack of capture is more severe. The dispensings of these subjects are paid by Medicare and therefore are not seen in the commercial claims databases even though these patients are present in the commercial database. Commercial claims databases coverage of subjects aged 65 years and older is limited to subjects that are commercially insured through a Medicare supplemental plan.

If the lack of capture was differential, that is, was related not only with the characteristics of the subjects but also with the outcome, then the direction of the bias that the exposure misclassification would introduce could be large and difficult to predict. As described previously, older subjects are more likely to have incomplete capture of dispensings than younger subjects. It has been shown that in the Medicare settings, sicker patients use $4 for generics programs more often than healthier patients 8, 9, so indeed, the misclassification could be differential depending on patient characteristics.

Because the use of $4 generics programs is becoming more popular, we expected that self‐paid prescriptions would be missing. Self‐paid dispensings, however, represented only 14% of the dispensings missed in PharMetrics Plus. It was unexpected that an important proportion of the missing dispensings (around 60%) was dispensings paid by a commercial insurer. Lack of coordination of benefits in subjects with more than one health insurance plan explains almost half of the missing prescriptions, and other factors, such as vouchers, also contributed.

Other studies have also found that claims databases fail to capture dispensings.^10^ These studies have used a diverse set of designs, data sources, and focus of interest. They include studies using commercial claims databases to compare the proportion of subjects exposed to a specific drug in different time periods to infer missing dispensings 11; using the Medical Expenditure Panel Survey to quantify the degree of use of low‐cost generic programs to infer the degree of missing dispensing in claims databases 12, 13; using self‐report medication use to assess lack of capture in claims databases 14; and examining the impact of drug samples provided by physician offices on drug exposure misclassification.^15^

The magnitude of missing dispensings in those studies, not surprisingly, varies from 10% to 36%.^11^, ^12^, ^14^ The present study offers the advantages of quantifying in millions of subjects the magnitude of missing dispensings and does it directly and objectively, because it does not rely on self report data. It also depicts a more comprehensive picture of the degree of missing dispensings because we report the missing dispensings at a patient and dispensing level.

Our findings suggest that studies in which one arm is a medication and the comparator arm is a non‐user of that medication would be more prone to exposure misclassification bias than when the comparator is an active medication because missing dispensings can be expected to affect both arms in the latter, but not in the former. Studies have shown that the choice of a comparator substantially influences risk estimation^16^ and that studies that used non‐users as comparators could lead to biased estimates.^17^ Increased susceptibility to exposure misclassification bias can be added to the list of reasons as to why that study design can be problematic. One approach to mitigate the lack of complete capture of dispensings is the inclusion of active comparators.

Another potential solution to the lack of complete dispensing capture would be to supplement the exposure data in commercial claims databases with data from pharmacy databases that capture all transactions including cash transactions. In particular, cash transactions have been recognized as important for understanding patient behavior and have been associated with doctor and pharmacy shopping for opioids and stimulants.^18^, ^19^

Sensitivity analyses to assess the susceptibility of the results to bias due to missing prescriptions and to adjust the estimates to account for exposure misclassification bias can also be used 20, 21. The findings of this study can guide researchers to elicit the probability of exposure misclassification needed to conduct deterministic or probabilistic sensitivity analyses.

We included four medication classes that are commonly used, and the pattern of lack of capture in PharMetrics Plus was similar in all the medications. We see no reasons for other medications to differ, or for other claims databases to differ, but this is unknown. Studies using identical study design, but conducted in different databases, can and do generate different results.^22^

The comparisons of the number of dispensings between PharMetrics Plus and LRx were conducted at the therapeutic class level; comparisons at the specific product level may produce different results.

While dispensings in PharMetrics Plus had been adjudicated, it is possible that further adjudications may occur at a later time so the magnitude of the lack of capture of dispensings could decrease overtime. It could also be argued that the missing dispensings in PharMetrics Plus represent reversals (i.e., the patient did not pick the medication or refused to pay for the medication—the co‐pay) and are not truly dispensings. IMS estimates that no more than of 1% of the transactions in LRx could be reversals. Therefore, the potential presence of reversals in LRx cannot explain the study findings.

The linkage between PharMetrics Plus and LRx has a false positive rate (i.e., believing there is a patient match when it is not really a match) of 1–2%; this rate is too small to explain the lack of capture of all dispensing in 19% of the subjects.

In summary, researchers need to be aware of the failure of commercial claims databases to completely capture dispensings. The lack of capture goes beyond the known limitation of not capturing cash transactions. It is substantial and can introduce substantial misclassification bias.

## Conflict of Interest

M. Soledad Cepeda and Daniel Fife are employees of Janssen Research and Development. The manuscript makes no reference to any commercial product. Michel Denarié, Dan Bradford, Stephanie Roy, and Yingli Yuan are IMS Health employees. IMS Health is the owner of the databases used in the study.

**Key Points**

- Commercial claims databases do not provide a complete picture of all medication dispensings
- When compared to an open longitudinal prescription data set, commercial claims databases missed 25% of dispensings.
- Commercial claims databases identified all dispensings to 68% of patients, identified some dispensings but missed ≥1 dispensing to 13% of subjects, and missed all the dispensings to 19% of the subjects.
- The lack of capture of dispensings in commercial claims databases is substantial, goes beyond cash transactions, and may introduce substantial misclassification bias.
- Augmenting health insurance plan claims with longitudinal prescription data offers a more complete picture of true drug utilization.

## Ethics Statement

The New England Institutional Review Board determined that this type of research is not human research.

## Supporting information


**Supplement Table 1.** Codes used to identify opioids, diuretics, anti‐platelet medications, anti‐coagulants.
**Supplement Table 2. Flow of subjects**
