# Supplementary material for: Quantification of missing prescriptions in commercial claims databases: results of a cohort study
Source: Pharmacoepidemiol Drug Saf. 2017 Jan 25;26(4):386–92. doi: 10.1002/pds.4165 (PMC5396298; doi:10.1002/pds.4165)
Supplement: Supplementary file 1 — Supplement Table 1. Codes used to identify opioids, diuretics, anti‐platelet medications, anti‐coagulants. Supplement Table 2. Flow of subjects [file PDS-26-386-s001.docx]

**Supplement Table 1**

Codes used to identify opioids, diuretics, anti-platelet medications, anti-coagulants.

| Medication Class | 10 digit generic product identifier* | Products Included |
| --- | --- | --- |
| Opioids | 6510002500, 6510002510, 6510003010, 6510003510, 6510005010, 6510005510, 6510005520, 6510005570, 6510007510, 6510008010, 6510009110, 6520001000, 6599170210, 6599170250 | BUPRENORPHINE, FENTANYL, FENTANYL CITRATE, HYDROCODONE BITARTRATE, HYDROCODONE-ACETAMINOPHEN, HYDROCODONE-IBUPROFEN, HYDROMORPHONE HCL, METHADONE HCL, MORPHINE SULFATE, MORPHINE SULFATE BEADS, MORPHINE-NALTREXONE, OXYCODONE HCL, OXYMORPHONE HCL, TAPENTADOL HCL |
| Diuretics | 3720001000, 3720002000, 3720003000, 3720008000, 3740001000, 3750001010, 3750002000, 3750003000, 3760002000, 3760002500, 3760004000, 3760005000, 3760005500, 3760006000, 3799000210, 3799000220, 3799000230 | AMILORIDE & HYDROCHLOROTHIAZIDE, AMILORIDE HCL, BUMETANIDE, CHLOROTHIAZIDE, CHLORTHALIDONE, ETHACRYNIC ACID, FUROSEMIDE, GLYCERIN, HYDROCHLOROTHIAZIDE, INDAPAMIDE, METHYCLOTHIAZIDE, METOLAZONE, SPIRONOLACTONE, SPIRONOLACTONE & HYDROCHLOROTHIAZIDE, TORSEMIDE, TRIAMTERENE, TRIAMTERENE & HYDROCHLOROTHIAZIDE |
| Anti-Coagulants | 8310002020, 8310002021, 8310101010, 8310101410, 8310102010, 8310108010, 8310303010, 8320001000, 8320003020, 8333403000, 8337001000, 8337003020, 8337006000 | APIXABAN, DALTEPARIN SODIUM, DANAPAROID SODIUM, DESIRUDIN, DICUMAROL, EDOXABAN TOSYLATE, ENOXAPARIN SODIUM, FONDAPARINUX SODIUM, HEPARIN SODIUM (BOVINE), HEPARIN SODIUM (PORCINE), RIVAROXABAN, TINZAPARIN SODIUM, WARFARIN SODIUM |
| Anti-Platelets | 6410001000, 6410990202, 8515002010, 8515003000, 8515301000, 8515303000, 8515306010, 8515306011, 8515551600, 8515578030, 8515601010, 8515802010, 8515806010, 8515808010, 8515847000, 8515990220 | ABCIXIMAB, ANAGRELIDE HCL, ASPIRIN, ASPIRIN BUFFERED (AL HYDROXIDE-MAG HYDROXIDE), ASPIRIN-DIPYRIDAMOLE, CILOSTAZOL, CLOPIDOGREL BISULFATE, DIPYRIDAMOLE, EPTIFIBATIDE, PRASUGREL HCL, TICAGRELOR, TICLOPIDINE HCL, TIROFIBAN HCL, TIROFIBAN HCL IN SODIUM CHLORIDE, VORAPAXAR SULFATE |

***Medi-Span electronic drug file, © Wolters Kluwer.**

**Supplement Table 2 Flow of subjects**

| **Step #** | **Description** | **Number of Patients** | **Number of patients in LRx** |
| --- | --- | --- | --- |
| 1 | Patients identified in PharMetrics Plus with continuous enrollment (medical and pharmacy) from 4/2014-3/2015 | 19,480,497 |  |
| 2 | Patients from step 1 that match to a unique patient ID in LRx | 12,898,712 |  |
| 3 | Patients from step 2 with a dispensing in LRx for any opioids, diuretics, antiplatelet medications or anticoagulants.  (Patients with Rxs in multiple markets are double counted) | 1,528,878 | 7,165,304 |
| 4 | Patients from step 3 with more Rxs in LRx than PharMetrics Plus in at least 1 of the 4 markets  (Patients with Rxs in multiple markets are double counted) | 1,426,498 | 6,594,154 |
|  | Diuretics | 426,696 | 2,567,019 |
|  | Opioids | 821,205 | 2,944,767 |
|  | Antiplatelet medications | 96,626 | 572,901 |
|  | Anticoagulants | 81,971 | 509,467 |
